# Access to Primary Care and Internet Searches for Walk-In Clinics and Emergency Departments in Canada: Observational Study Using Google Trends and Population Health Survey Data

**DOI:** 10.2196/13130

**Published:** 2019-11-18

**Authors:** Joseph Ssendikaddiwa, Ruth Lavergne

**Affiliations:** 1 Faculty of Health Sciences Simon Fraser University Burnaby, BC Canada

**Keywords:** internet, ambulatory care facilities, emergency departments, primary health care, health services accessibility

## Abstract

**Background:**

Access to primary care is a challenge for many Canadians. Models of primary care vary widely among provinces, including arrangements for same-day and after-hours access. Use of walk-in clinics and emergency departments (EDs) may also vary, but data sources that allow comparison are limited.

**Objective:**

We used Google Trends to examine the relative frequency of searches for walk-in clinics and EDs across provinces and over time in Canada. We correlated provincial relative search frequencies from Google Trends with survey responses about primary care access from the Commonwealth Fund’s 2016 International Health Policy Survey of Adults in 11 Countries and the 2016 Canadian Community Health Survey.

**Methods:**

We developed search strategies to capture the range of terms used for walk-in clinics (eg, urgent care clinic and after-hours clinic) and EDs (eg, emergency room) across Canadian provinces. We used Google Trends to determine the frequencies of these terms relative to total search volume within each province from January 2011 to December 2018. We calculated correlation coefficients and 95% CIs between provincial Google Trends relative search frequencies and survey responses.

**Results:**

Relative search frequency of walk-in clinic searches increased steadily, doubling in most provinces between 2011 and 2018. Relative frequency of walk-in clinic searches was highest in the western provinces of British Columbia, Alberta, Saskatchewan, and Manitoba. At the provincial level, higher walk-in clinic relative search frequency was strongly positively correlated with the percentage of survey respondents who reported being able to get same- or next-day appointments to see a doctor or a nurse and inversely correlated with the percentage of respondents who reported going to ED for a condition that they thought could have been treated by providers at usual place of care. Relative search frequency for walk-in clinics was also inversely correlated with the percentage of respondents who reported having a regular medical provider. ED relative search frequencies were more stable over time, and we did not observe statistically significant correlation with survey data.

**Conclusions:**

Higher relative search frequency for walk-in clinics was positively correlated with the ability to get a same- or next-day appointment and inversely correlated with ED use for conditions treatable in the patient’s regular place of care and also with having a regular medical provider. Findings suggest that patient use of Web-based tools to search for more convenient or accessible care through walk-in clinics is increasing over time. Further research is needed to validate Google Trends data with administrative information on service use.

## Introduction

### Primary Care, Emergency Department, and Walk-In Clinic Use in Canada

This paper used Google Trends to explore search patterns for walk-in clinics and emergency departments (EDs) in Canada. High-quality, accessible primary care is central to the effectiveness and efficiency of health care systems [1,2]. In Canada, primary care is intended to be the first point of contact with the health care system and provides access to referred health care services and coordination of care [3]. However, access to primary care continues to be a challenge for many Canadians, with Canada ranking below average in cross-national surveys of primary care access. For example, according to the Commonwealth International Health Policy Survey for adults in 11 countries, Canada ranks below average on timely access to care with only 43% of Canadians reporting to have gotten a same- or next-day appointment at their regular place of care and only 34% reporting to have access to after-hours care without going to the emergency room [4]. In the absence of access to care with a regular provider, patients may turn to EDs or walk-in clinics as alternatives.

EDs provide crucial lifesaving health care and are designed to provide episodic care to patients with injuries or acute illnesses, though patients may also turn to them for primary care services that are otherwise inaccessible or inconvenient [5-7]. The ED setting is also a costly setting to deliver chronic and continuing care and may lead to mishandling or poor attention to upstream social and chronic health issues of frequent ED users within the episodic ED setting [8]. Walk-in clinics also provide primary care services to patients without an appointment [6]. They may be a more convenient alternative for patients with another regular source of primary care [9] or the only source of care for patients without a regular primary care provider. As with EDs, there are concerns that walk-in clinics may disrupt continuity of care or neglect preventive health care [6] and lead to duplication of services, failure to manage complex care needs, and higher costs [9]. Understanding patterns of walk-in clinic use is therefore important in planning primary care policy more broadly.

In Canada, primary care is financed and organized provincially, and provinces have undertaken quite varied approaches to primary health care reform, including changing models of care, physician remuneration, and after-hours access programs [10-12]. The province of Ontario has adopted the use of various models of interprofessional care, including family health networks and family health teams [13]. Western provinces and Quebec have implemented different organizational reforms, including family medicine groups, local services networks, medical homes, and primary care networks [14-17]. The proportion of clinical payments through capitated and salaried payment models is higher in Ontario, eastern provinces, and the territories, whereas the western provinces and Quebec have a higher proportion of fee-for-service payments [14,15,18]. Walk-in clinic practice may be more common under a fee-for-service model as physicians are paid per service without ongoing responsibility for specific patients. Provinces also have varying mechanisms for after-hours coverage. Most have a toll-free number that connects patients to a registered nurse who can provide advice and direct patients to after-hours care [10,14]. Under capitated models, physicians may receive negation if their patients access services from another primary care provider, including walk-in clinics. This variability in the organization and delivery of primary care may shape availability and use of walk-in clinic services, and also use of EDs for conditions that could be treated in primary care.

Despite the importance of monitoring the use of walk-in clinics and EDs, there are no comparable nationwide data sources to track their use. Although each province collects data on use of primary care services, there are no standard approaches to identify walk-in practices [19,20]. The Canadian Institute of Health Information collects some standardized data on ED use through the National Ambulatory Care Reporting System. Though coverage is increasing, this is currently available for only a subset of facilities in Canada [21].

### Use of Google Trends in Health Services and Policy Research

Access to health care starts before the clinic door, and patients may use search engines like Google to navigate access to needed care, including walk-in clinics and EDs. They may use Google searches to identify sources of care, confirm location, check hours, or obtain other information such as wait times. Though not a direct measure of health care use, relative search frequency may provide insight into patterns of care seeking across provinces and over time.

The application of internet data provided by tools like Google Trends in health services research has potential to complement and extend the data that presently exist, collected primarily through survey and administrative sources. For example, internet data have been used quite extensively in epidemiology and public health research for the surveillance of infectious disease outbreaks, determining patterns and seasonality of disease incidence, and examining information seeking about health conditions [22,23]. Application of Google Trends in health care and health services research has been more limited but has included information seeking on addiction treatment programs and correlation between Google searches for dementia and Alzheimer diseases, and outpatient visits [21,24-29]. A systematic review of studies using Google Trends in health care research found a 7-fold increase in publications from 2009 to 2013, with 27% of the studies on infectious diseases, 24% on mental health and substance use, 16% on other noncommunicable diseases, and 33% on general population behavior [22]. However, to our knowledge, no studies have used Google Trends to examine searches for walk-in clinic or ED services.

In this paper, we used Google Trends to compare search frequencies for walk-in clinics and EDs across provinces in Canada and over time (2011-2018). We compared observed search patterns with survey data capturing access to primary care.

## Methods

### Data

#### Google Trends

Google Trends provides an index of the volume of searches by geographic location. The search index reflects search volume for each search term in a given geographic region divided by the total search volume in that region, over a defined time period [30,31].

We developed search strategies to capture the range of terms used for walk-in clinics and EDs across Canadian provinces. These were based on both researcher knowledge and additional suggested terms supplied by the Google Trends interface. Multiple search terms can be combined with a plus sign (+) that denotes OR [32], and results include searches containing either term. The resulting searches were as follows:

- Search 1: walk-in clinics: “walk-in clinic + walk-in clinic + urgent care clinic + medical clinic + after-hours clinic”
- Search 2: EDs: “emergency department + emergency room + ER”

Though *emergency department* is commonly abbreviated as *ED,* it was not possible to separate relevant searches from those seeking information on *erectile dysfunction*, also commonly abbreviated as *ED*. It was therefore excluded from the final search.

We downloaded province-level search indices over the period from January 1, 2011, to December 31, 2018. We began our study period in 2011 as a change in geographic assignment was implemented by Google effective January 1, 2011. As only 5 comparisons could be made per batch within the Google Trends interface, the Canada-wide search was included in all batches and used to standardize search frequency for each province by dividing each monthly provincial search frequency by the Canadian average over the entire study period. The reported *relative search frequency* values in this manuscript, therefore, reflect search frequency for each province and month relative to the Canadian average over the entire study period.

We developed French-language search terms, but the Google Trends interface limits the number of terms that can be included. It was not possible to combine both French and English terms within 1 batch, and so we excluded Quebec from analysis, as search frequencies would not be comparable. As the populations of Yukon, Northwest, and Nunavut Territories are small, Google Trends does not return a search index, and we could not include them in analysis.

#### National Survey Data

No data exist tracking ED and walk-in clinic visits consistently over time or across provinces, so it was not possible to compare information from Google sources to other administrative data sources directly capturing service use. To explore the plausibility of search results, we compared information from Google Trends with province-level, cross-sectional survey data capturing patient-reported primary care access. We used data from 2 national surveys to capture primary care access. The Commonwealth Fund’s International Health Policy Survey of Adults is conducted annually over the phone among nationally representative samples of noninstitutionalized adults ages 18 years and older in 11 high-income countries. [4]. The following variables were analyzed:

- Same- or next-day access: percentage of respondents who were able to get an appointment to see a doctor or a nurse the same or next day the last time they were sick;
- After-hours access: percentage of respondents who thought it was very easy or somewhat easy to get medical care in the evenings, weekends, or holidays without going to the hospital ED;
- Use of ED for condition treatable at a regular place of medical care: percentage of respondents who reported that the last time they went to the hospital ED, it was for a condition that they thought could have been treated by providers at usual place of care if they had been available.

The Canadian Community Health Survey (CCHS) is conducted annually among noninstitutionalized people 12 years of age and above across all 10 provinces and 3 territories. An area frame is used to select the CCHS target population aged 18 years and over, and the Canadian Child Tax Benefit frame used to select participants aged 12 to 17 years [33]. We analyzed the following variable from the CCHS Survey:

- Access to a regular health care provider: percentage of respondents with a regular health care provider (a health professional that one sees or talks to regularly when they need care or advice on health) [33].

### Analysis

All search frequencies are expressed as a ratio of each monthly provincial value to the Canadian average over the study period (2011-2018). Plots of these data are included in Multimedia Appendices 1 and 2. To identify provinces with higher and lower search volumes, and those that experienced more dramatic changes over the study period, we used linear regression to calculate the intercept (model-predicted relative search frequency in January 2011), slope, average over the study period, and relative increase from January 2011 to December 2018 (based on model-predicted values at these time points). We report the Pearson correlation coefficients (*r*) and associated 95% CIs for the relationship between each province’s relative search frequencies for walk-in clinics and EDs over the 8-year study period, and provincial survey results (expressed as a percentage within each province). We interpret correlation coefficients between 0.7 and 1.0 as indicating variables that are highly correlated, between 0.5 and 0.7 as moderately correlated, and between 0.3 and 0.5 as being weakly correlated.

## Results

On average, over the study period from 2011 to 2018, walk-in clinic searches were most frequent in the western provinces of British Columbia, Alberta, Saskatchewan, and Manitoba. Relative search frequencies for walk-in clinics increased steadily, doubling in most provinces between 2011 and 2018 (Table 1). ED searches were most frequent in Prince Edward Island and Manitoba. There was no consistent pattern of change over time in ED searches among provinces.

Provincial variation was also evident in survey data covering topics related to primary care access (Table 2). The percentage of respondents reporting that they were able to get a same- or next-day appointment tended to be higher in the western provinces (Table 2). After-hours access was highest in Alberta and Ontario. Eastern provinces of Newfoundland and Labrador, Prince Edward Island, Nova Scotia, and New Brunswick reported highest use of ED for conditions treatable at a regular place of medical care. Western provinces of British Columbia, Alberta, Saskatchewan, and Manitoba had lower percentages of adults reporting having a regular medical doctor.

We observed a strong positive correlation between walk-in clinic relative search frequencies and being able to get a same- or next-day appointment (*r*=0.77, 95% CI 0.23 to 0.95; Table 3). We observed a strong negative correlation between walk-in clinic relative search frequency and use of ED for conditions treatable at the patient’s regular place of care (*r*=−0.71, 95% CI −0.93 to −0.09), and having a regular health care provider (*r*=−0.89, 95% CI −0.98 to −0.56; Table 3). We observed no significant correlations between ED relative search frequencies and survey data (Table 3).

**Table 1:** Intercepts and trends for Google Trend relative search frequencies by province.

Province	Google Trends walk-in clinic searches^a^				Google Trends emergency department searches^a^			
	Intercept (January 2011)	Slope (monthly change)	Average over study period	Change over study period	Intercept (January 2011)	Slope (monthly change)	Average over study period	Change over study period
British Columbia	0.99	0.013	1.59	2.25	0.98	0.004	1.18	1.39
Alberta	1.01	0.012	1.55	2.13	0.69	0.007	1.01	1.97
Saskatchewan	0.92	0.014	1.59	245	0.79	0.002	0.85	1.24
Manitoba	0.87	0.011	1.38	2.20	0.79	0.013	1.39	2.56
Ontario	0.64	0.007	1.00	2.03	0.82	0.004	1.00	1.46
New Brunswick	0.50	0.002	0.53	1.38	1.40	0.005	1.00	1.34
Nova Scotia	0.73	0.009	1.13	2.18	0.92	0.004	1.10	1.41
Prince Edward Island	1.48	−0.004	1.06	0.74	0.10	0.028	1.58	27.60
Newfoundland and Labrador	0.51	0.007	0.82	2.30	0.71	–0.003	1.01	0.60

^a^Search frequencies are expressed relative to the Canadian average search frequency over the study period from January 2011 to December 2018 (ie, a value of 1.00 is equal to the Canadian average, values less than 1 are lower than the Canadian average, and values greater than 1 are higher than the Canadian average).

**Table 2:** Provincial percentage responses from the 2016 Commonwealth Fund International Health Policy Survey of Adult and the 2016 Canadian Community Health Survey.

Province	Same- or next-day access, %^a^	After-hours access, %^b^	Use of ED^c^ for condition treatable at a regular place of medical care, %^d^	Has a regular health care provider, %^e^
British Columbia	44	27	36	83
Alberta	48	42	30	82
Saskatchewan	49	32	43	81
Manitoba	47	34	40	85
Ontario	44	40	44	90
New Brunswick	33	35	52	90
Nova Scotia	34	26	48	90
Prince Edward Island	30	25	60	89
Newfoundland and Labrador	34	16	49	90

^a^Same- or next-day access: percentage of respondents who were able to get an appointment to see a doctor or a nurse the same or next day the last time they were sick.

^b^After-hours access: percentage of respondents who thought it was very easy or somewhat easy to get medical care in the evenings, weekends, or holidays without going to the hospital ED.

^c^ED: emergency department.

^d^Use of ED for condition treatable at a regular place of medical care: percentage of respondents who reported that the last time they went to the hospital ED, it was for a condition that they thought could have been treated by the doctors or staff at the place where they usually get medical care if they had been available.

^e^Has a regular health care provider: percentage of respondents who have a regular health care provider (a health professional that one sees or talks to regularly when they need care or advice on health).

**Table 3:** Correlation between Google Trends relative search frequency (average 2011-2018) and provincial percentage responses from the 2016 Commonwealth Fund International Health Policy Survey of Adult and the 2016 Canadian Community Health Survey.

Correlations	Walk-in clinic		Emergency department	
	*r*	95% CI	*r*	95% CI
Same- or next-day access	0.77	0.23 to 0.95	−0.34	−0.82 to 0.42
After-hours access	0.23	−0.51 to 0.78	−0.24	−0.78 to 0.51
Use of ED^a^ for condition treatable at a regular place of medical care	−0.71	−0.93 to −0.09	0.37	−0.39 to 0.83
Has a regular health care provider	−0.89	−0.98 to −0.56	0.17	−0.56 to 0.75

^a^ED: emergency department.

## Discussion

### Principal Findings

Walk-in clinic relative search frequency was highest in western provinces and is increasing over time across Canada. Provinces that had high walk-in clinic relative search frequencies reported greater same- or next-day access to care and lower use of ED for conditions treatable at the patient’s regular place of medical care. The percentage of patients with a regular medical doctor is also lower in provinces with high relative search frequency for walk-in clinics.

Findings may suggest that challenges accessing primary care have contributed to increasing patient use of Web-based tools to search for more convenient and easily accessible care through walk-in clinics [34]. The degree to which the rapid increase in searches for walk-in clinics reflects increased availability of this option relative to other sources of primary care [3], a preference for the convenience of walk-in style practice [9], or another explanation cannot be determined from our data.

The strong positive correlation between walk-in clinic searches and same- or next-day access and strong negative correlations with ED use for treatable condition and having a regular medical provider lend credibility to the Google Trend results. Walk-in clinics may make it easier to get timely services without going to ED [35]. However, this could raise concerns about discontinuity and poor coordination of care [6] as more patients also lack regular medical providers. It may be that people lack regular medical providers because physicians are not taking more regular patients but are instead choosing walk-in style practice [19].

ED search frequency varies by province, but there is no clear pattern east to west nor consistent pattern of increase or decrease over the study period. In the province of Manitoba, ED closures were a prominent topic in the media over the study period, which may explain high relative search frequencies in this province and would in no way correspond to service use. We did not observe correlations between ED search frequency and survey data, including ED use for condition treatable by provider at a regular place of care. It is likely that Google Trends less directly reflects ED service use than walk-in clinic use. Most people already know the location of major hospitals and attached EDs, which are open 24/7. As such, people may not need to search through Google to find their location or operating hours. Walk-in clinics are more numerous, may open and close in different locations, and may have fluctuating hours of operation and so service use may be better reflected in search data for walk-in clinics.

### Limitations

This study is subject to a number of limitations. First, Google searches can only partially reflect patterns of care seeking. Not all searches correspond to actual use, and not all use would be preceded by a search. It is plausible that the use of Google as a tool in accessing health care also differs across patients and provinces, independent of realized service use. We have no ability to validate Google Trends data against administrative data sources collected over time. Comparison with survey data adds credibility to search frequency for walk-in clinics, but this analysis is limited by the sample size for cross-provincial comparisons. This study builds on an existing body of literature that has confirmed the plausibility of Google Trends search results as public health indicators by correlating Google Trends data with other sources [36].

The correlations made are at the ecological level and require caution in interpretation. People who answered the surveys are not the same as those doing the searches. Survey data include only those who choose to respond, and search data only reflects Canadians who use the internet [37]. We were unable to disaggregate search data by demographic characteristics or other characteristics related to both search behavior and primary care access. It may be that internet users more commonly use walk-in clinics, but this would be true across provinces and over time and would not explain the province-level relationships observed.

### Conclusions

Findings from Google Trends are consistent with survey information about the province-level access to primary care, and may offer some insight into how the organization of primary care differs across provinces. Findings suggest that patient use of Web-based tools to search for more convenient or accessible care through walk-in clinics is increasing. Further research is needed to validate Google Trends data with administrative information on service use.

## Appendix

Multimedia Appendix 1 Relative search frequency for walk-in clinics from 2011-2018 across nine Canadian provinces.

Multimedia Appendix 2 Relative search frequency for emergency departments from 2011 to 2018 across nine Canadian provinces.

## Abbreviations

CCHS: Canadian Community Health Survey
ED: emergency department
