# Supplementary figures and images for: Access to Primary Care and Internet Searches for Walk-In Clinics and Emergency Departments in Canada: Observational Study Using Google Trends and Population Health Survey Data
Source: JMIR Public Health Surveill. 2019 Nov 18;5(4):e13130. doi: 10.2196/13130 (PMC6913775; doi:10.2196/13130)

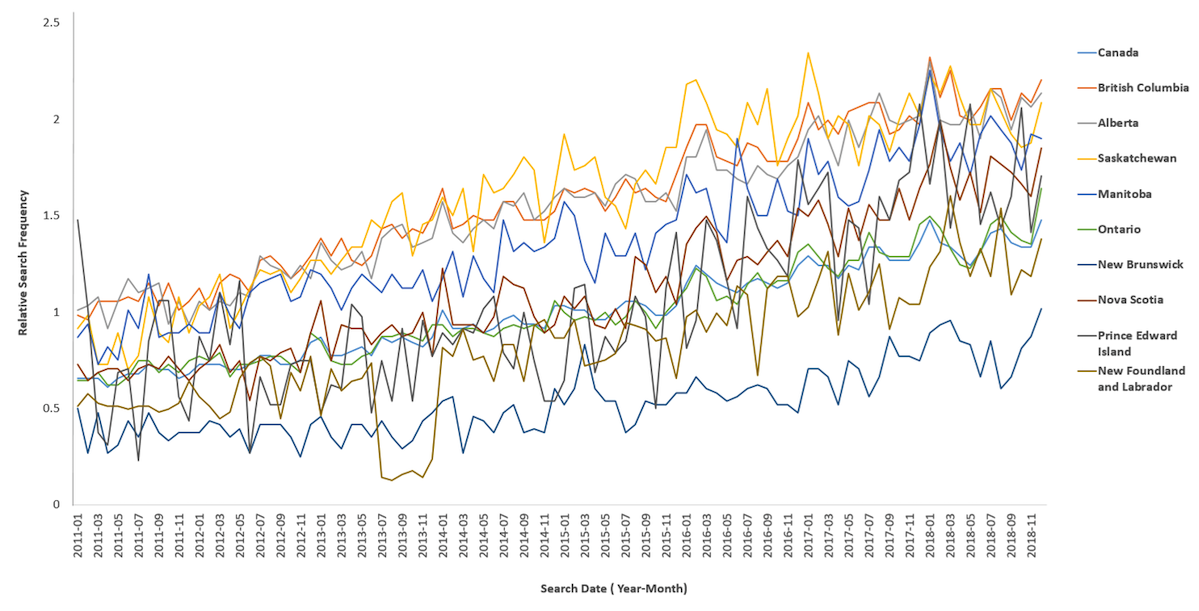

Supplement: Multimedia Appendix 1 [file publichealth_v5i4e13130_app1.png]

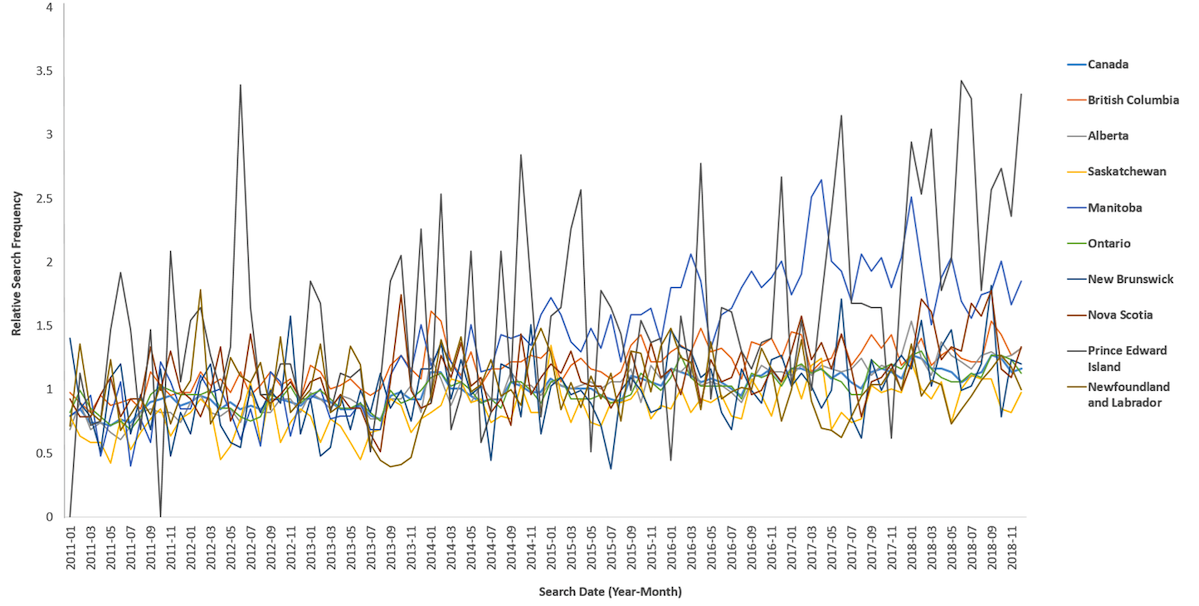

Supplement: Multimedia Appendix 2 [file publichealth_v5i4e13130_app2.png]
